# Immediate postpartum family planning utilization and its associated factors among postpartum women in Ethiopia: a systematic review and meta-analysis

**DOI:** 10.3389/fgwh.2023.1095804

**Published:** 2023-08-22

**Authors:** Mulualem Silesh, Tesfanesh Lemma Demisse, Birhan Tsegaw Taye, Tebabere Moltot, Moges Sisay Chekole, Girma Wogie, Fetene Kasahun, Solomon Adanew

**Affiliations:** Department of Midwifery, Asrat Woldeyes Health Science Campus, Debre Berhan University, Debre Berhan, Ethiopia

**Keywords:** immediate postpartum, family planning, postpartum, utilization, Ethiopia

## Abstract

**Background:**

Family planning integration in areas where women contact the healthcare system routinely is essential for addressing the high unmet need for family planning among postpartum women and reducing the risk of short interpregnancies. Immediate postpartum family planning (IPPFP) is an integrated service, and opportunities exist for women by providing family planning (FP) counseling and contraceptives as part of care following childbirth within 48 h. Therefore, this review aimed to assess the pooled estimate of immediate postpartum family planning utilization and its associated factors in Ethiopia.

**Method:**

Electronic databases were used to conduct an extensive search of all published studies, and the digital library was used to identify any unpublished studies. An observational study that reports the prevalence/magnitude and/or associated factors/predictors/determinants of IPPFP utilization in Ethiopia was included. Data were extracted on the Microsoft Excel spreadsheet and analyzed using STATA Version 11. A random-effects model was applied to determine the pooled prevalence of immediate postpartum family planning utilization with a 95% confidence interval (CI). Inverse variance (*I*^2^) was used to identify the presence of heterogeneity, and a funnel plot and Egger's test were used to check the presence of publication bias. Subgroup analysis was conducted based on the sample size, region, and year of study to identify the source of heterogeneity.

**Result:**

Of 15 primary studies, the overall pooled prevalence of immediate postpartum family planning utilization among postpartum women in Ethiopia was 21.04% (95% CI: 13.08, 29.00). Received counseling on FP [OR: 3.59; 95% CI (1.84, 7.01; *P* < 0.001), having a positive attitude toward FP [OR: 3.2; 95% CI (1.23, 8.35); *P* = 0.017], and partner support to use FP [OR: 5.85; 95% CI (1.12, 30.54; *P* = 0.036) were significant predictors of immediate postpartum family planning utilization.

**Conclusion:**

Generally, IPPFP utilization in Ethiopia was insufficient. Therefore, to enhance the utilization, integrating FP counseling at all maternal service care points, strengthening community awareness to develop a favorable attitude toward family planning, and promoting partner involvement in family planning counseling are essential.

**Systematic Review Registration:**

https://www.crd.york.ac.uk/PROSPERO/display_record.php?RecordID=239053, identifier: CRD42021239053.

## Introduction

Improving the availability of contraceptives is essential to achieving the 2030's Sustainable Development Goals (SDGs) (1) and universal access to reproductive health services during pregnancy, childbirth, and the first year after delivery (2), which helps in decreasing the risk of premature birth, low birth weight, fetal and neonatal death, and adverse maternal health outcomes (3).

Postpartum family planning (PPFP) integration in areas where women contact the healthcare system routinely, such as during antenatal care, labor and delivery, postnatal care, immunization, and child healthcare, is essential for addressing the high unmet need for family planning (FP) among postpartum women and reducing the risk of short interpregnancies (4). Immediate postpartum family planning (IPPFP) is an integrated service, and opportunities exist for women by providing FP counseling and contraceptive as part of care following childbirth within 48 h (5, 6).

According to the World Health Organization (WHO) technical consultation committee for better maternal and child health outcomes, an interval of at least 2 years following a live birth is recommended before attempting another pregnancy (7). Infant and under-five child mortality would decrease by 10% and 21%, respectively, in those women who waited 2 years between giving birth and getting pregnant again (8).

Postpartum women have a greater unmet need for contraceptives than nonpostpartum women (6). Globally, 225 million women desire modern contraception to delay or prevent future pregnancy, but they do not have access (9). The demographic health survey data from 57 countries indicated that 62% of women in the first year after birth have an unmet need for FP (10). A study conducted in low- and middle-income countries showed that the unmet need for FP among postpartum women ranged from 25% to 96% (11). Pregnancies that happened within the first year of the delivery were mostly unplanned (12) and associated with increased risk of adverse maternal, perinatal, infant, and child health outcomes, ranging from stillbirth, small-for-gestational-age, low birthweight to neonatal and maternal morbidity and mortality (13, 14).

Maternal health problems related to pregnancy and childbirth remain a major global concern and leading causes of morbidity and mortality among reproductive-age women (9). As WHO's 2019 report, 810 women die every day from preventable causes related to pregnancy and childbirth, of which 94% of all maternal mortality occurs in low- and lower-middle-income countries (15). The prevalence of unintended pregnancy is still high and the most challenging issue within the domain of maternal and child health and women's reproductive health globally (16, 17). According to the WHO report from the estimated 210 million pregnancies worldwide, 44% of pregnancies were unintended and around 59% of pregnancies ended by abortion (18).

Although there are some fragmented primary studies on family planning utilization during the immediate postpartum period, most of them focused only on the postpartum intrauterine contraceptive device utilization in Ethiopia. Also, the determinants were variable among these studies. However, the pooled national level by addressing all options of IPPFP utilization was unknown. Therefore, this meta-analysis aimed to estimate the pooled prevalence of immediate postpartum family planning utilization and its associated factors in Ethiopia by considering the WHO Medical eligibility criteria for contraceptives-2015 for postpartum during the immediate postpartum period. The result of this study will provide important input for policymakers and healthcare providers about IPPFP utilization and will enforce them to design evidence-based strategies for improving IPPFP utilization in Ethiopia.

### Objectives of the review

• To determine the pooled prevalence of immediate postpartum family planning uptake in Ethiopia
• To identify the predictors of immediate postpartum family planning uptake in Ethiopia

## Methods

### Study design, data source, and search strategy

This systematic review and meta-analysis was conducted to estimate the pooled prevalence of IPPFP utilization and its associated factors in Ethiopia under the guidelines of the Preferred Reporting Items for Systematic Reviews and Meta-analyses (PRISMA) statement (19, 20). We checked to see whether there were any similar ongoing systematic reviews and meta-analyses in the *PROSPERO* database, but no such studies had been conducted before. Then, the review protocol was registered on the PROSPERO database with registration number *CRD42021239053*. Finally, a standard systematic review and meta-analysis reporting PRISMA checklist was used to present the findings.

Furthermore, four authors (MS, TD, GW, and TM) explored all relevant studies were searched in the following databases; PubMed, Google Scholar, African Journal of Online (AJOL), HINARI, Scopus, Science Direct, Excerpta Medica database (EMBASE), DOAJ, and Web of Science. Google and the organization's website were used to search the gray literature and unpublished research studies. Studies were searched by using the full title (immediate postpartum family planning utilization and associated factors in Ethiopia) and the following searching keywords or terms; “Immediate postpartum Family planning,” “Postpartum family planning,” “Postpartum Intrauterine Contraceptive Device,” “Postpartum Intrauterine Device,” “PPIUCD,” “PPIUD,” “Uptake,” “Utilization,” “Use,” “Determinants,” “Predictors,” “associated factors,” “immediate postpartum period,” and “Ethiopia.” To find any further missed studies, the reference lists of all included published and unpublished studies were reviewed. All fields and MeSH (Medical Subject Headings) terms with Boolean operators (“OR” and/or “AND”) were used to search studies in the advanced PubMed search engine (Additional File S1).

### Eligibility criteria

Both published and unpublished observational studies (cross-sectional and case–control), which report the prevalence/magnitude and/or associated factors/predictors/determinants of IPPFP utilization in Ethiopia, were included. Also, this review included studies done until July 30,2022. Studies with a different outcome of interest/operational definitions and inaccessibility of full text were excluded.

### Outcome measurement, study selection, and quality assessment

IPPFP utilization was considered when a postpartum woman was using any of the postpartum contraceptive options (progesterone-only pills, implant, intrauterine devices, and permanent FP method) within 48 h of delivery. After studies were searched from different international databases and organization's websites, studies were screened by using the following criteria (duplication, relevancy, accessibility of full text, and outcomes of interest). Finally, the quality of each study was assessed using the standard quality assessment tool [Newcastle–Ottawa scale (NOS)] (21) and by four authors (MS, SA, BTT, and MSC) independently using the following components: selection, comparability, and outcome, which were graded by five, two, and three, respectively. Any disagreements between the four authors during quality appraisal were resolved by the other two authors (FK and TLD) through discussion and re-evaluation of selected studies. For analysis, only the primary studies with a medium score (fulfilling 50% of the quality evaluation criteria) and above were included (22) (Additional File S2).

#### Data extraction process

Data were extracted using a data extraction format prepared in a Microsoft Excel 2010 spreadsheet by three authors (MS, BTT, and MSC). This form includes the first author's name, study year, study area, region, study design, sample size, prevalence of IPPFP utilization, and AOR with a 95% CI for factors associated with the utilization of IPPFP. By involving a fourth author (TLD), differences at the time of data extraction were solved through discussion and consensus.

### Data synthesis and statistical analysis

Data were extracted using Microsoft Excel 2010 spreadsheet and analyzed using STATA Version 11 software. Because high heterogeneity across studies was identified using inverse variance (*I*^2^) statistics with its corresponding *P*-value (*I*^2^ = 98.6%, *P *< 0.001) (23–25), a random-effects model was applied to determine the pooled prevalence of IPPFP utilization. Also, subgroup analysis was performed based on the sample size, year of study, and region to identify the source of heterogeneity across studies. A funnel plot and Egger's test were used to check publication bias (26). A *P*-value of less than 0.05 was used to declare the statistical significance of publication bias (27). Due to this, the publication bias for meta-analysis results was addressed using the Duval and Tweedie nonparametric trim and fill analysis by random-effects analysis (28, 29). A factor associated with IPPFP uptake in at least two primary studies was included in the meta-analysis. The results were presented using texts, tables, and forest plots with effect and 95% CI measures.

## Results

### Study selection

**Figure 1:**
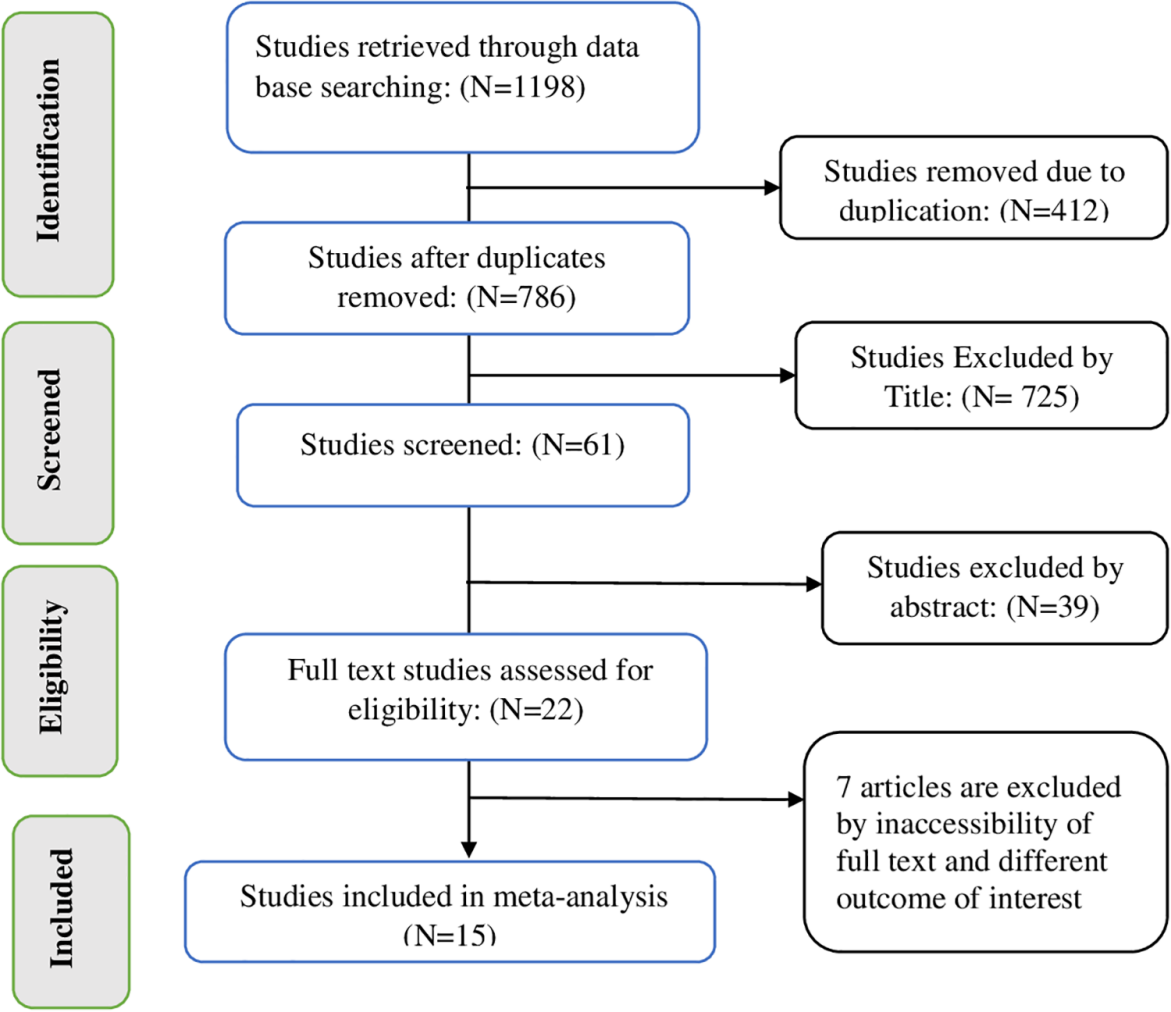
PRISMA flow diagram of included studies to estimate the pooled prevalence of immediate postpartum family planning utilization and its associated factors among women in Ethiopia, 2022.

A total of 1,198 studies were searched from different international databases and Ethiopian universities' institutional repositories using different searching strategies. The EndNote 7 reference manager was used to screen all retrieved studies. As a result, 412 studies were removed due to duplication. Then, 771 studies were excluded due to unrelated titles, abstract inaccessibility of full text, and different outcomes of interest. Finally, 15 studies that met the inclusion criteria were considered for the meta-analysis (Figure 1).

### Characteristics of included studies

Fifteen primary studies were included in this systematic review and meta-analysis, of which 13 were cross-sectional studies, while two were case–control studies with a sample size ranging from 182 to 884 (30, 31). Regarding the region in which the study was conducted, four articles were from Amhara (31–34), five from SNNP (35–39), four from Addis Ababa (31, 40–42), and two from the Oromia (43, 44) region with the year of study ranging from 2016 to 2020. The highest prevalence of IPPFP utilization was reported by Arero et al. (53.2%) (44), which was done in the Oromia region, while the lowest IPPFP utilization was reported in the Amhara region by Hagos et al. (3.3%) (30) (Table 1).

**Table 1:** Descriptive summary of 15 studies included to estimate the pooled prevalence of immediate postpartum family planning utilization in Ethiopia.

Author	Year study	Region	Study design	Outcome (FP methods)	Study design	Sample size	Prevalence
Geda et al. (40)	2019	AA	Cross-sectional	PPIUCD	Cross-sectional	286	26.6
Demissie et al. (41)^a^	2019	AA	Cross-sectional	ALL IPPFP	Cross-sectional	586	12.97
Mohammed (42)^a^	2019	AA	Cross-sectional	PPIUCD	Cross-sectional	286	26.6
Belayihun et al. (31)	2019	AA	Cross-sectional	LARC	Cross-sectional	884	39
Hagos et al. (30)	2018	Amhara	Cross-sectional	PPIUCD	Cross-sectional	182	3.3
Melkie et al. (32)	2019	Amhara	Cross-sectional	PPIUCD	Cross-sectional	423	4
Assefaw et al. (33)	2019	Amhara	Case Control	PPIUCD	Case Control	420	NA
Silesh et al. (34)	2020	Amhara	Cross-sectional	ALL IPPFP	Cross-sectional	396	21.3
Usso et al. (43)	2020	Oromia	Cross-sectional	LARC	Cross-sectional	530	18.5
Arero et al. (44)	2016	Oromia	Cross-sectional	LARC	Cross-sectional	393	53.2
Gebremedhin et al. (35)	2019	SNNP	Cross-sectional	PPIUCD	Cross-sectional	452	14
Gejo et al. (36)	2018	SNNP	Cross-sectional	ALL IPPFP	Cross-sectional	368	13.6
Tefera et al. (37)	2016	SNNP	Cross-sectional	PPIUCD	Cross-sectional	310	21.6
Mohammed et al. (38)	2019	SNNP	Case–control	PPIUCD	Case Control	510	NA
Tariku et al. (39)	2019	SNNP	Cross-sectional	LARC	Cross-sectional	418	25.4

AA, Addis Ababa; SNNP, South Nations, Nationalities, and Peoples; LARC, long-acting reversible contraceptive; NA, not applicable.

^a^ Unpublished studies.

#### Prevalence of immediate postpartum family planning uptake

**Figure 2:**
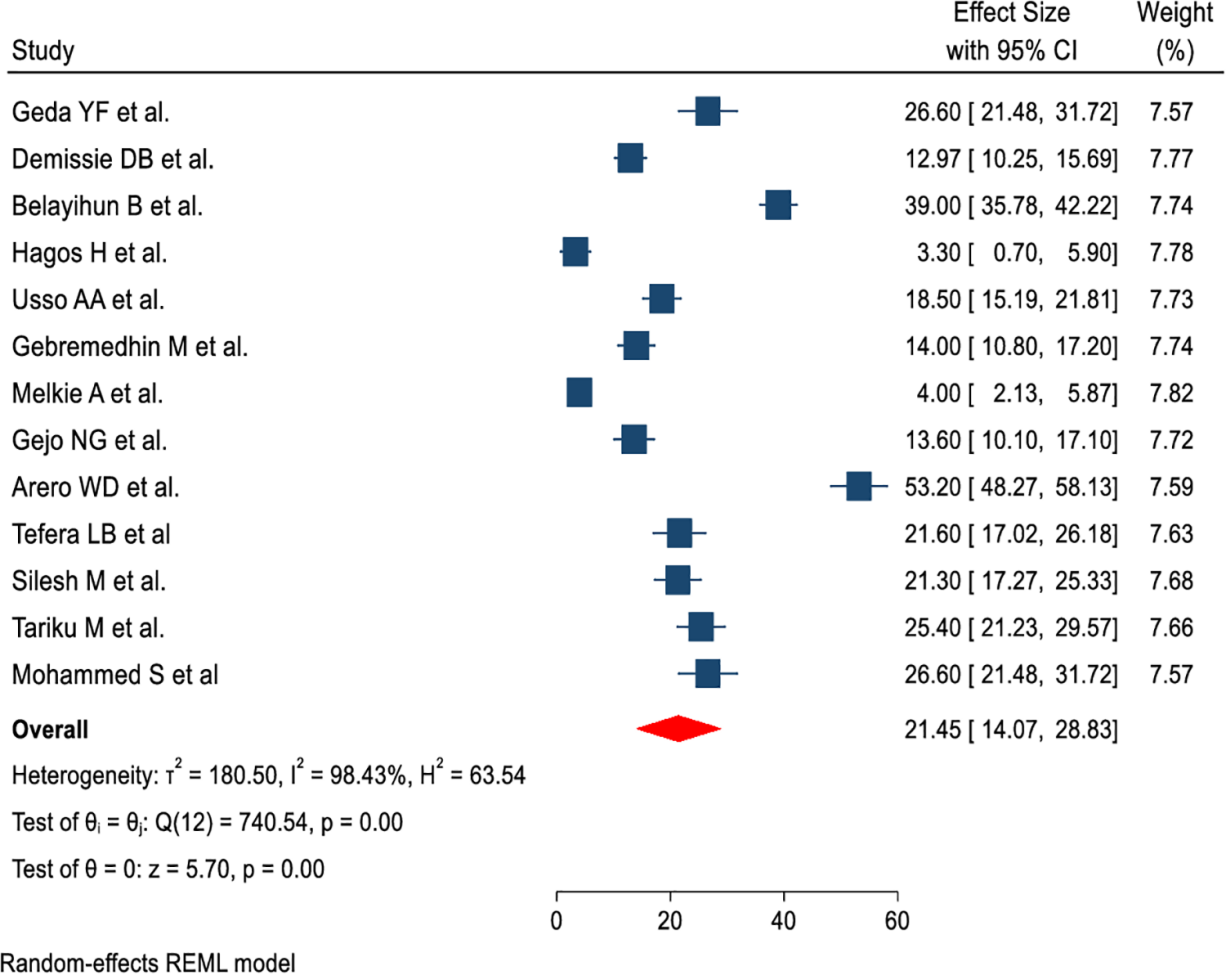
Forest plot of pooled prevalence of immediate postpartum family planning utilization among postpartum women in Ethiopia.

A total of 13 (11 published and two unpublished) studies with 5,514 postpartum women were included in this review to estimate the pooled prevalence of IPPFP uptake. Accordingly, this study revealed high heterogeneity across the studies, as evidenced by *I*^2^ statistics (*I*^2^ = 98.43%, *P* < 0.001); the random-effects model was applied to estimate the pooled prevalence of IPPFP utilization. Therefore, the pooled prevalence of IPPFP utilization among postpartum women in Ethiopia was 21.45% (95% CI: 14.07, 28.83) (Figure 2).

### Publication bias and heterogeneity

**Figure 3:**
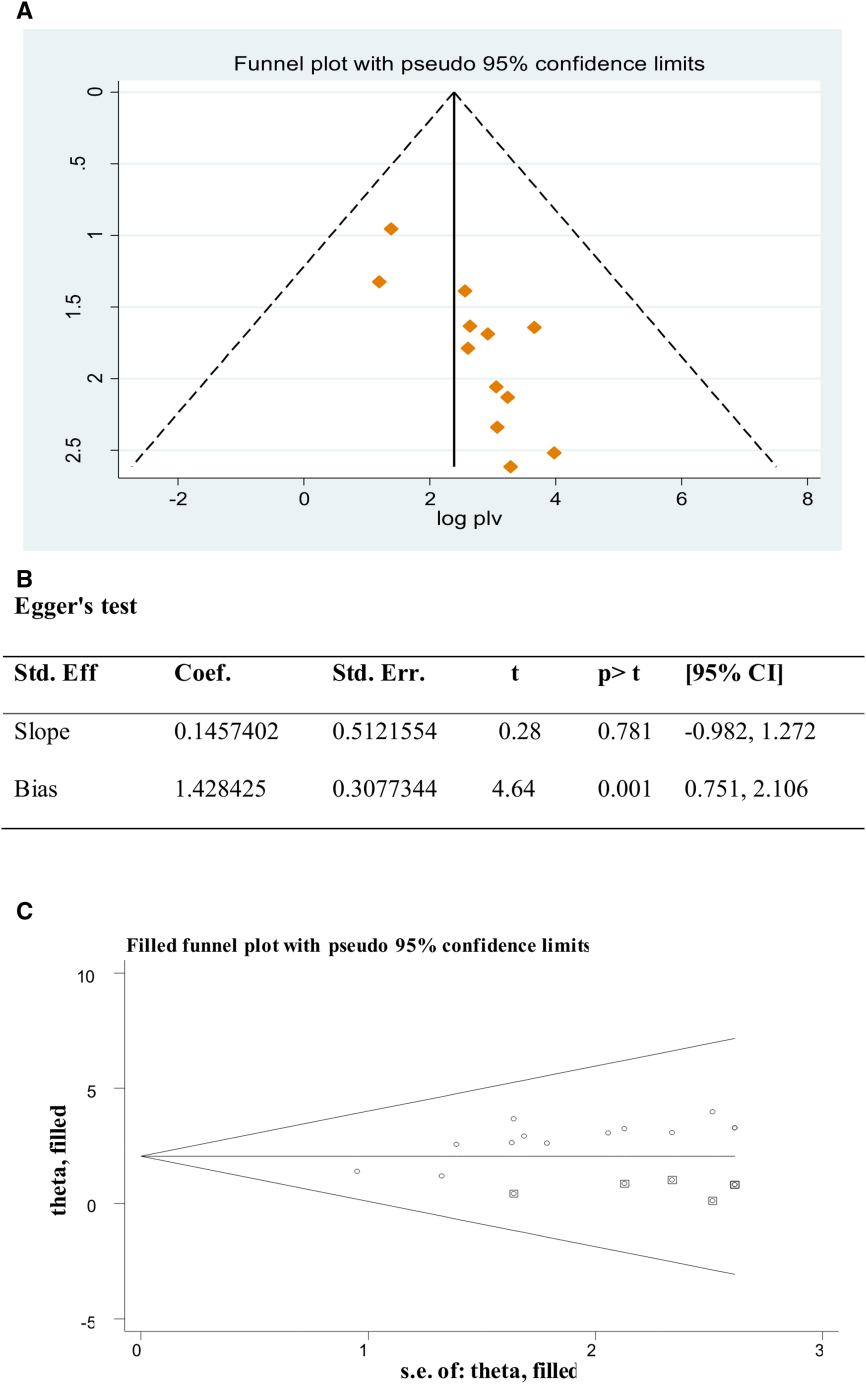
(A) Funnel plot to test publication bias of 13 studies. (B) Egger’s test, (C) result of the trim and fill analysis.

A funnel plot and Egger's test (26) were used to check publication bias. A *P*-value of less than 0.05 was used to declare the statistical significance of publication bias (27). Accordingly, the funnel plots' results revealed an asymmetrical pattern, which suggests that the included studies have a publishing bias (Figure 3A). In addition, Egger's regression test revealed that studies had publication bias (*P*-value = 0.001 (Figure 3B). Therefore, the Duval and Tweedie nonparametric trim and fill analysis using the random-effects method was conducted to deal with the publication bias for meta-analysis results (28, 29). As a result, after 6 studies were filled, 19 studies were included to resolve the publication bias across studies by trim and fill analysis to produce the pooled estimate of IPPFP utilization of 2.05 (1.24, 2.85) (Figure 3C).

#### Subgroup analysis and sensitivity analysis

Significant heterogeneity was observed among included primary studies. Therefore, subgroup analysis was conducted based on the sample size, region, and year of study to identify the source of heterogeneity. Accordingly, the overall prevalence of IPPFP uptake was found to be high among studies conducted with <400 sample size [23.52 (11.84, 35.20)], in the Oromia region [35.75 (1.74, 69.75)], and in the year 2016 [37.3 (6.33, 68.27)] (Table 2).

**Table 2:** Subgroup analysis of immediate postpartum family planning utilization in Ethiopia (*n* = 13).

Subgroup	Number of studies	Prevalence (95% CI)	*I*^2^ and *P*-value
Sample size			
<400	7	23.52 (11.84, 35.20)	(97.2%, *P* ≤ 0.001)
≥400	6	18.71 (9.22, 28.2)	(95.5%, *P* ≤ 0.001)
*Overall*	13	21.25 (14.29, 28.20)	(96.3%, *P* ≤ 0.001)
Year of study			
2020	2	19.83 (15.70, 23.97)	(0%, *P* = 508)
2019	7	20.99 (11.56, 30.43)	(95.7%, *P* ≤ 0.001)
2018	2	8.19 (1.89, 18.27)	(92.2%, *P* ≤ 0.001)
2016	2	37.3 (6.33, 68.27)	(97.5%, *P* ≤ 0.001)
*Overall*	13	21.25 (14.29, 28.20)	(96.3%, *P* ≤ 0.001)
Region			
Addis Ababa	4	26.11 (15.30, 36.92)	(91.8%, *P* ≤ 0.001)
Amhara	3	8.87 (1.47, 16.27)	(93.5%, *P* ≤ 0.001)
Oromia	2	35.75 (1.74, 69.75)	(98%, *P* ≤ 0.001)
SNNP	4	18.41 (12.84, 23.97)	(74.4%, *P* = 0.008)
*Overall*	13	21.25 (14.29, 28.20)	(96.3%, *P* ≤ 0.001)

CI, confidence interval; SNNP, south nations, nationalities, and peoples.

A leave-one-out sensitivity analysis was performed to check the effect of individual studies on the pooled estimate of IPPFP utilization. In the sensitivity analysis, the pooled prevalence of IPPFP utilization was observed to be low at 10.02% (3.90%, 25.72%) and high at 15.28% (5.43%, 42.98%) when the studies of Belayihun et al. and Melkie et al. were omitted, respectively (Table 3).

**Table 3:** Sensitivity analysis of immediate postpartum family planning utilization in Ethiopia (*n* = 13).

Study omitted	Year of study	Estimated prevalence (95% CI)
Geda et al.	2019	10.85 (4.33, 27.19)
Demissie et al.	2019	10.95 (4.19, 28.56)
Belayihun et al.	2019	10.02 (3.90, 25.72)
Hagos et al.	2018	13.20 (5.03, 34.67)
Usso et al.	2020	10.71 (4.18, 27.43)
Gebremedhin et al.	2019	10.94 (4.26, 28.09)
Melkie et al.	2019	15.28 (5.43, 42.98)
Gejo et al.	2018	10.99 (4.32, 28.06)
Arero et al.	2016	10.57 (4.21, 26.52)
Tefera et al.	2016	10.86 (4.32, 27.33)
Silesh et al.	2020	10.78 (4.27, 27.28)
Tariku et al.	2019	10.71 (4.24, 27.06)
Mohammed et al.	2019	10.85 (4.33, 27.19)

CI, confidence interval.

#### Determinants of immediate postpartum family planning uptake

From the nine identified factors from primary studies, counseling on FP, attitude toward FP, and having partner support to use FP showed statistically significant associations with IPPFP utilization in this meta-analysis. However, the age of women, ever heard about FP, knowledge of FP, discussion with the partner, planned status of the pregnancy, and plan to have another child showed no statistically significant association with IPPFP utilization (Additional File S3).

**Figure 4:**
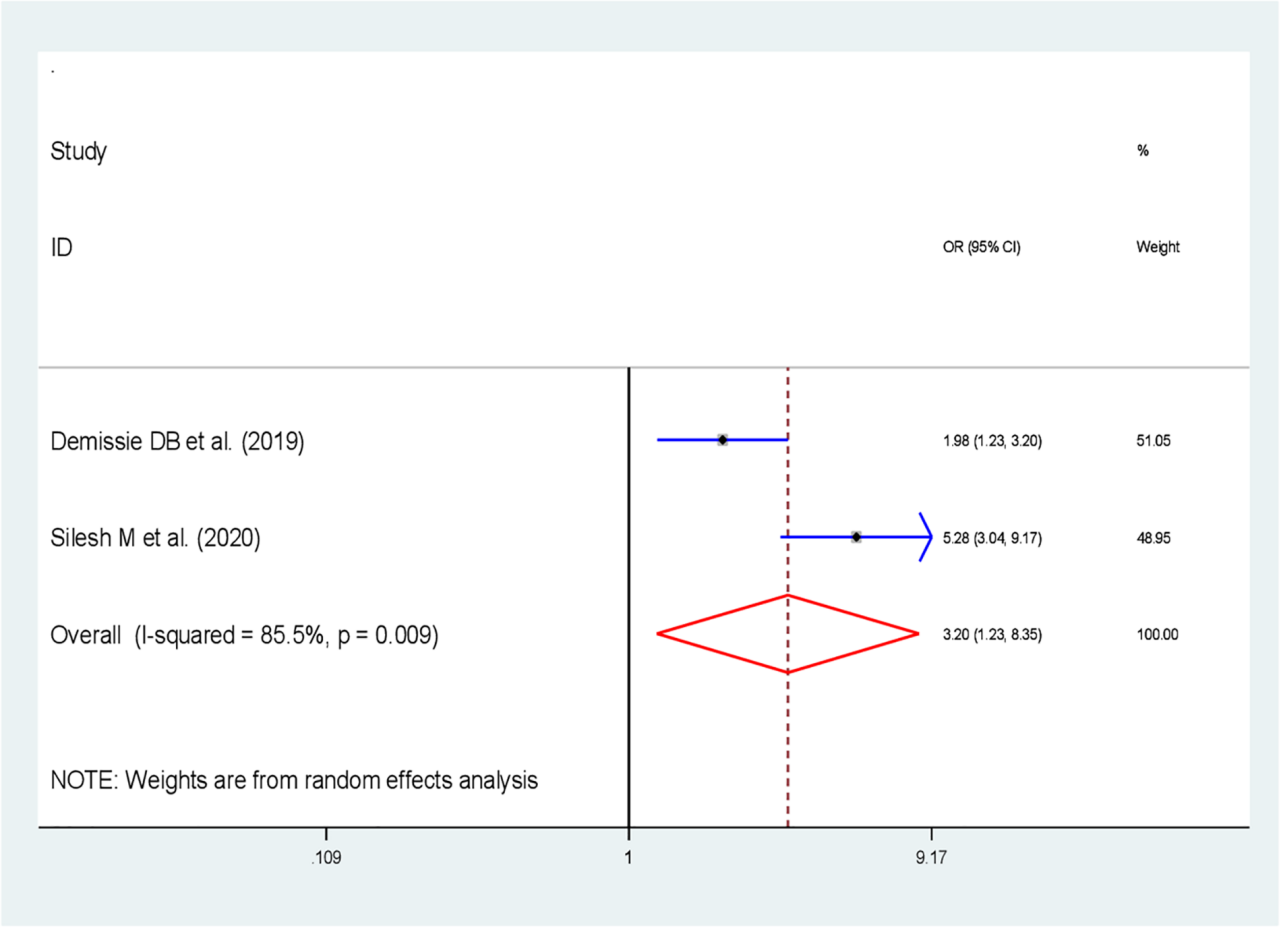
Forest plot showing the association between attitudes toward FP employed status and immediate postpartum family planning utilization in Ethiopia.

The pooled effect of attitude toward FP on IPPFP utilization among women during the immediate postpartum period was evaluated using two primary studies (34, 41). The result of this study revealed that women's attitude toward FP was significantly associated with IPPFP utilization, and the likelihood of utilizing IPPFP was 3.2 times higher among those women who had a positive attitude toward FP than their counterparts [OR: 3.2; 95% CI (1.23, 8.35); *P = *0.017], with heterogeneity (*I*^2^ = 85.5%, *P*-value = 0.009) (Figure 4).

**Figure 5:**
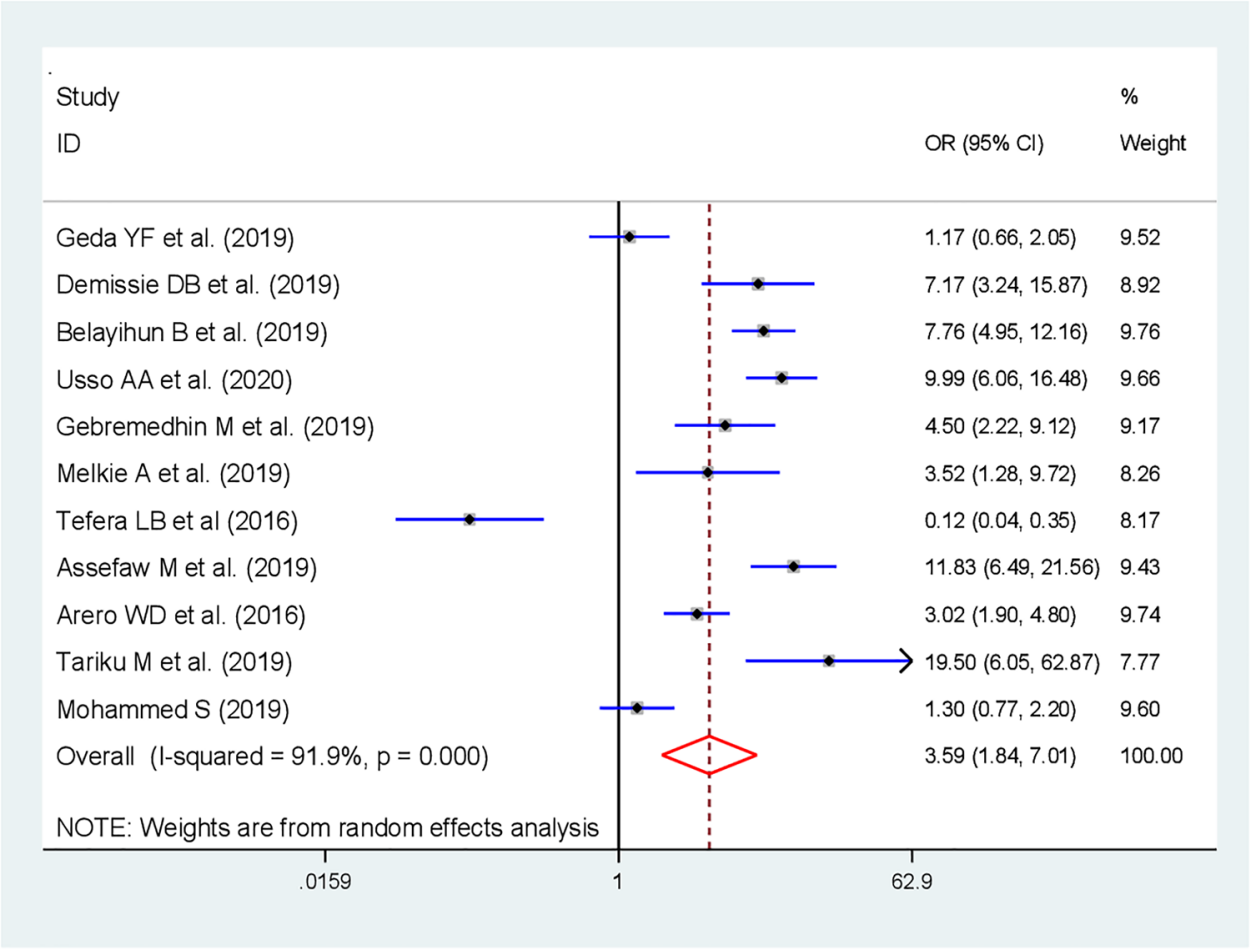
Forest plot showing the association between counseling on FP and immediate postpartum family planning utilization in Ethiopia.

Eleven studies were included in determining the pooled effects of FP counseling status on IPPFP utilization (31, 32, 44, 33, 35, 37, 39–43). The findings revealed that FP counseling status was significantly associated with the utilization of IPPFP; those women who received FP counseling were 3.6 times more likely to utilize IPPFP than women who were not counseled on FP [OR: 3.59; 95% CI (1.84, 7.01; *P* < 0.001)] with heterogeneity (*I*^2^ = 91.9%, *P*-value <0.001) (Figure 5).

**Figure 6:**
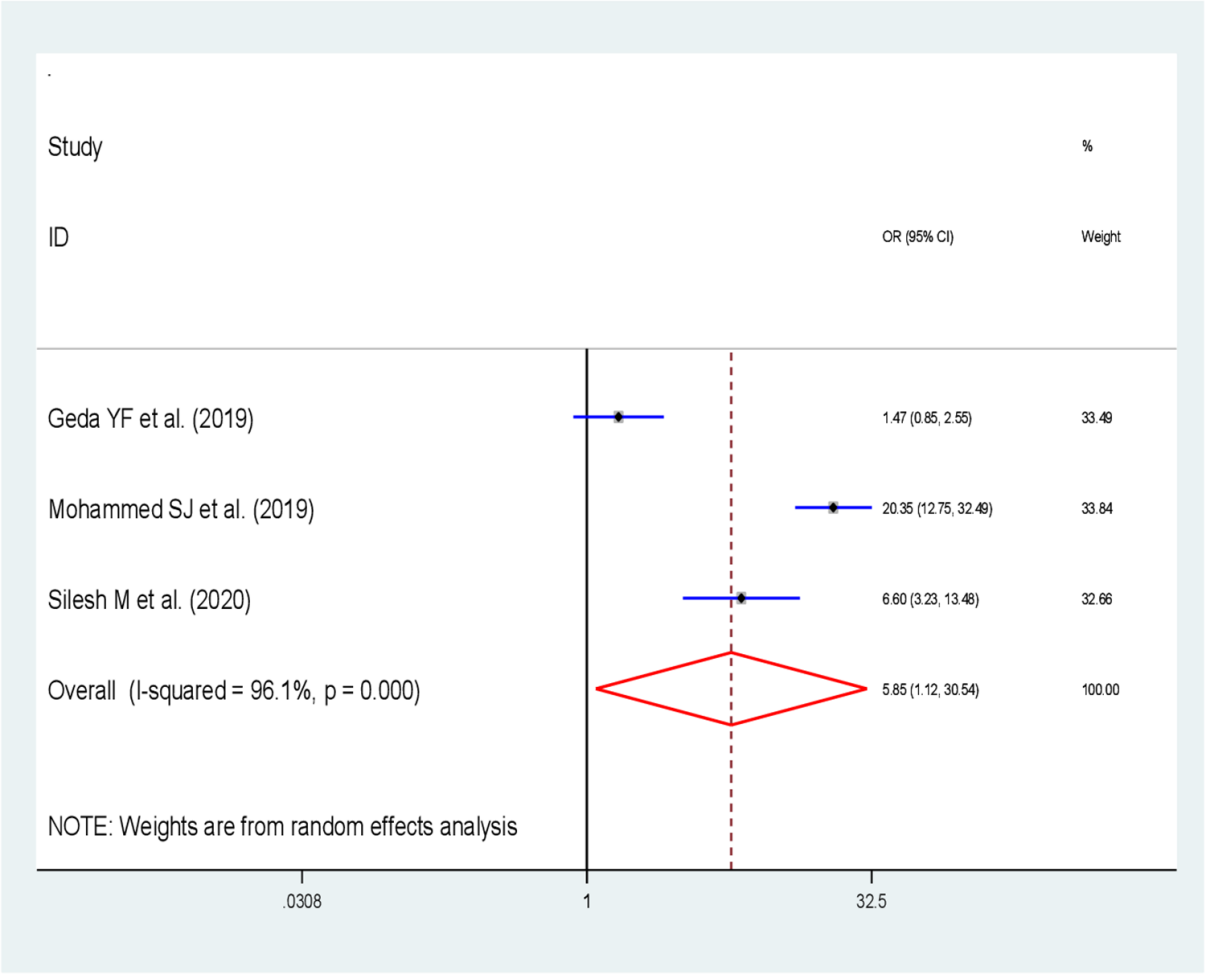
Forest plot showing the association between partner support and immediate postpartum family planning utilization in Ethiopia.

Furthermore, the effect of partner support to use FP on IPPFP utilization was assessed by using three studies (34, 38, 40). The finding of this study revealed that the pooled effect of having partner support to use FP was significantly associated with the utilization of IPPFP; postpartum women who had partner support to use FP were almost six times more likely to use IPPFP than their counterparts [OR: 5.85; 95% CI (1.12, 30.54; *P = *0.036)], with heterogeneity (*I*^2 ^= 96.1%, *P*-value <0.001) (Figure 6).

## Discussion

This systematic review and meta-analysis aimed to assess the pooled prevalence and associated factors of IPPFP utilization in Ethiopia. To the best of our knowledge, this meta-analysis is a first of its kind in determining the national prevalence and significant factors of IPPFP utilization in Ethiopia, which is used as input for policymakers, healthcare providers, and other stakeholders to design evidence-based strategies to strengthen IPPFP utilization.

The overall pooled prevalence of IPPFP uptake among postpartum women in Ethiopia was 21.04% (95% CI: 13.08, 29.00). This finding is comparable with a study done in Ethiopia on PPIUCD (21.63%) (22) but lower than a systematic review and meta-analysis conducted in low- and middle-income countries (41.2%) (45) and Ethiopia (45.44%–45.79%) (46, 47). The possible reason for this variation could be attributed to variations in the study setting, study period, and accessibility of health facilities. In addition to this, variation in sociocultural and women's health-seeking behavior between the studies settings might contribute to this difference.

In the present review and meta-analysis, subgroup analysis was done based on the region and year of study, indicating no variations in the uptake of IPPFP among the region and year of study in Ethiopia. Accordingly, the highest utilization of PPIUCD was observed in Oromia at 35.75% (1.74, 69.75) (43, 44) and the studies conducted in 2016 at 37.29% (6.33, 68.27) based on the region, study population, and year of study, respectively. The observed variation could be due to regional religious and cultural differences. In addition, IPPFP uptake improved from time to time; the difference in residence and inclusion criteria of primary studies on information exposure could be the possible reason for the discrepancies in the uptake of IPPFP by year of study.

In this meta-analysis, counseling status was positively associated with IPPFP utilization. Women who received counseling on FP were four times more likely to utilize IPPFP compared to their counterparts. This is consistent with studies conducted in four countries (India, Nepal, Sri Lanka, and Tanzania) (48) and Ethiopia (47). Also, a study done in Ethiopia revealed that women who had been counseled were 3.05 times more likely to utilize PPIUCD than those who had not been counseled about PPIUCD (22). This might be because counseling could significantly help women to have accurate information about family planning, which further changes the negative attitudes of women toward family planning and minimize myths and misconceptions related to family planning.

The odds of uptake of IPPFP were three times higher among women who have a positive attitude toward FP than women who have a negative attitude toward FP. This might be because one's attitude toward a particular activity is crucial to how something will be carried out in practical situations.

Moreover, the finding of this study revealed that women who had partner support to use FP were six times more likely to utilize IPPFP than women who have not had partner support. A similar finding was also reported by the study conducted in Ethiopia; PPIUD uptake was 11.48 times higher in those women who had husband/partner support than those who had no support (22).

This study has some limitations. The lack of studies from most regions may affect the generalizability of this study. Furthermore, significant heterogeneity was detected across studies, which undermines the pooled estimate of IPPFP utilization. Subgroup analysis was done based on the sample size, region, and year of study. However, the possible source of heterogeneity was not identified.

## Conclusion

The overall prevalence of IPPFP utilization was low in Ethiopia. Being counseled on FP, having a positive attitude toward FP, and having partner support to use FP were significantly associated with IPPFP utilization. Therefore, to enhance immediate postpartum family planning utilization, integrating FP counseling at all maternal service care points, strengthening community awareness to develop a favorable attitude toward family planning, and promoting partner involvement in family planning counseling are essential. Furthermore, for the researchers, it is better to assess the factors related to health facilities and healthcare providers on IPPFP utilization.

## Data Availability

The datasets presented in this study can be found in online repositories. The names of the repository/repositories and accession number(s) can be found in the article/**Supplementary Material**.
