# Supplementary material for: Immediate postpartum family planning utilization and its associated factors among postpartum women in Ethiopia: a systematic review and meta-analysis
Source: Front Glob Womens Health. 2023 Aug 22;4:1095804. doi: 10.3389/fgwh.2023.1095804 (PMC10478094; doi:10.3389/fgwh.2023.1095804)
Supplement: Supplementary file 1 [file Table1.docx]

**Additional file 1:** Searching strategies from databases to assess the pooled estimate of immediate postpartum family planning uptake and its associated factors in Ethiopia

| **Databases** | **Searching terms** | **Number of studies** | **Searching period** |
| --- | --- | --- | --- |
| PubMed/ MEDLINE | **((((((Uptake) OR (Utilization)) OR (Utilisation)) OR (Use)) AND (((((((((((Immediate postpartum family planning) OR (postpartum family planning)) OR (Immediate postpartum)) OR (family planning)) OR (postpartum contraceptive)) OR (postpartum intrauterine device)) OR (postpartum)) OR (postpartum IUCD)) OR (PPIUCD)) OR (PPIUD)) OR (postpartum intrauterine contraceptive device))) AND (((Determinants) OR (Associated factors)) OR (Predictors))) AND (Ethiopia)** | 906 | Until 30/7/2022 |
| Google scholar | "Utilization" OR "Utilisation" OR "Uptake" AND "Immediate postpartum Family Planning " OR " Postpartum Intrauterine contraceptive Device" OR "Postpartum Intrauterine Device" OR "Postpartum IUCD" OR "PPIUCD" OR "PPIUD" AND "Associated Factors" OR "Predictors" OR "Determinants" AND "Ethiopia". | 124 |  |
| HINARI | ((Utilization) OR (Use) OR (Uptake) OR (Utilisation)) AND ((Immediate postpartum family planning) OR (Postpartum Intrauterine contraceptive Device) OR (Postpartum Intrauterine Device) OR (Postpartum IUCD) OR (PPIUCD) OR (PPIUD)) AND ((Associated Factors) OR (Predictors) OR (Determinants)) AND (Ethiopia) | 18 |  |
| Other databases | Uptake of Immediate Postpartum Family Planning and its associated factors in Ethiopia | 149 |  |
| Gray literature | Uptake of Immediate Postpartum Family Planning and its associated factors in Ethiopia | 1 |  |
| Total retrieved |  | 1198 |  |
| Finally, Eligible studies for review |  | 15 |  |
