# Supplementary material for: Immediate postpartum family planning utilization and its associated factors among postpartum women in Ethiopia: a systematic review and meta-analysis
Source: Front Glob Womens Health. 2023 Aug 22;4:1095804. doi: 10.3389/fgwh.2023.1095804 (PMC10478094; doi:10.3389/fgwh.2023.1095804)
Supplement: Supplementary file 2 [file Table2.docx]

**Additional file 2: *Newcastle-Ottawa Quality Assessment Scale to assess the use IPPFP among women in Ethiopia***

| **Studies** | **Selection** | | | | **Comparability** | **Outcome** | | **Total** |
| --- | --- | --- | --- | --- | --- | --- | --- | --- |
|  | Representativeness- (1) | Sample size- (1) | Non-respondents (1) | Ascertainment of the exposure (risk factor)- (2) | The subjects in different outcome groups are comparable - (2) | Assessment of the outcome - (2) | Statistical test- (1) |  |
| Geda YF et al. | * | * | * | * | ** | * | * | 8 |
| Demissie DB et al. | * | * | * | * | * | * | * | 7 |
| Belayihun B et al. | * | * | _ | * | ** | ** | * | 8 |
| Hagos H et al. | * | * | * | * | * | ** | _ | 7 |
| Usso AA et al. | * | * | * | ** | ** | * | * | 9 |
| Gebremedhin et al. | * | _ | * | * | * | ** | * | 7 |
| Melkie A et al. | * | * | * | ** | * | * | * | 8 |
| Gejo NG et al. | * | _ | _ | ** | ** | * | * | 7 |
| Arero WD et al. | * | * | * | * | ** | * | * | 8 |
| Tefera LB et al | * | * | * | ** | * | * | * | 8 |
| Silesh M et al. | * | * | * | ** | ** | * | * | 9 |
| Tariku M et al. | * | * | _ | ** | ** | * | * | 8 |
| Mohammed S. | * | * | * | ** | * | * | * | 8 |
| Mohammed SJ et al | * | * | * | * | * | ** | * | 8 |
| Assefaw M et al | * | * | * | * | * | * | * | 7 |

**Interpretation of the score**: Very Good Studies: 9-10 points, Good Studies: 7-8 points, Satisfactory Studies: 5-6 points and Unsatisfactory Studies: 0 to 4 points
