# Supplementary material for: Immediate postpartum family planning utilization and its associated factors among postpartum women in Ethiopia: a systematic review and meta-analysis
Source: Front Glob Womens Health. 2023 Aug 22;4:1095804. doi: 10.3389/fgwh.2023.1095804 (PMC10478094; doi:10.3389/fgwh.2023.1095804)
Supplement: Supplementary file 3 [file Table3.docx]

**Additional File 3:** Factors Not associated with Utilization of IPPFP in Ethiopia

| **Variables** | **Category** | **No. Studies** | **OR (95% CI)** | **P- value** | **I^2^ (%)** | **Heterogeneity test (P- value)** |
| --- | --- | --- | --- | --- | --- | --- |
| Age of women | <35 Vs ≥35 | 2 | 1.74 (0.275, 11.038) | 0.556 | 68.3 | 0.076 |
| Discussion with Partner | No Vs Yes | 3 | 2.58 (0.4, 16.561) | 0.319 | 97.3 | <0.001 |
| Ever heard about FP | No Vs yes | 2 | 4.95 (0.706, 34, 703) | 0.108 | 91.5 | 0.001 |
| Knowledge on FP | Poor Vs good | 3 | 0.68 (0.099, 4.696) | 0.697 | 96.9 | <0.001 |
| Plan to have another child | No Vs Yes | 3 | 1.26 (0.474, 3.365) | 0.641 | 89.9 | <0.001 |
| Planned status of the pregnancy | Unplanned vs Planned | 3 | 1.32 (0.922, 1.879) | 0.13 | 30.1 | 0.239 |
